# Cell walls of the dimorphic fungal pathogens *Sporothrix schenckii* and *Sporothrix brasiliensis* exhibit bilaminate structures and sloughing of extensive and intact layers

**DOI:** 10.1371/journal.pntd.0006169

**Published:** 2018-03-09

**Authors:** Leila M. Lopes-Bezerra, Louise A. Walker, Gustavo Niño-Vega, Héctor M. Mora-Montes, Gabriela W. P. Neves, Hector Villalobos-Duno, Laura Barreto, Karina Garcia, Bernardo Franco, José A. Martínez-Álvarez, Carol A. Munro, Neil A. R. Gow

**Affiliations:** 1 Lab. Cellular Mycology and Proteomics, Rio de Janeiro State University (UERJ), Rio de Janeiro, Brazil; 2 MRC Centre for Medical Mycology, Institute of Medical Sciences, University of Aberdeen, Aberdeen, United Kingdom; 3 Departamento de Biología, División de Ciencias Naturales y Exactas, Universidad de Guanajuato, Guanajuato, Gto., México; 4 Instituto Venezolano de Investigaciones Científicas, Centro de Microbiología y Biología Celular, Caracas, Venezuela; University of Tennessee, UNITED STATES

## Abstract

Sporotrichosis is a subcutaneous mycosis caused by pathogenic species of the *Sporothrix* genus. A new emerging species, *Sporothrix brasiliensis*, is related to cat-transmitted sporotrichosis and has severe clinical manifestations. The cell wall of pathogenic fungi is a unique structure and impacts directly on the host immune response. We reveal and compare the cell wall structures of *Sporothrix schenckii* and *S*. *brasiliensis* using high-pressure freezing electron microscopy to study the cell wall organization of both species. To analyze the components of the cell wall, we also used infrared and ^13^C and ^1^H NMR spectroscopy and the sugar composition was determined by quantitative high-performance anion-exchange chromatography. Our ultrastructural data revealed a bi-layered cell wall structure for both species, including an external microfibrillar layer and an inner electron-dense layer. The inner and outer layers of the *S*. *brasiliensis* cell wall were thicker than those of *S*. *schenckii*, correlating with an increase in the chitin and rhamnose contents. Moreover, the outer microfibrillar layer of the *S*. *brasiliensis* cell wall had longer microfibrils interconnecting yeast cells. Distinct from those of other dimorphic fungi, the cell wall of *Sporothrix* spp. lacked α-glucan component. Interestingly, glycogen α-particles were identified in the cytoplasm close to the cell wall and the plasma membrane. The cell wall structure as well as the presence of glycogen α-particles varied over time during cell culture. The structural differences observed in the cell wall of these *Sporothrix* species seemed to impact its uptake by monocyte-derived human macrophages. The data presented here show a unique cell wall structure of *S*. *brasiliensis* and *S*. *schenckii* during the yeast parasitic phase. A new cell wall model for *Sporothrix* spp. is therefore proposed that suggests that these fungi molt sheets of intact cell wall layers. This observation may have significant effects on localized and disseminated immunopathology.

## Author summary

Sporotrichosis was for many years attributed to a single etiological agent, *Sporothrix schenckii*. However, more recently, a new emerging pathogenic species, *Sporothrix brasiliensis*, has been shown to cause severe cases in humans and is associated with cat-transmitted sporotrichosis. In contrast, *S*. *schenckii* is related to a sapronosis and a benign human subcutaneous mycosis. The fungal cell wall is the first point of contact between the host and the pathogen and, therefore, plays an important role in pathogenesis. The data presented here demonstrate a novel cell wall structural organization for *S*. *brasiliensis* and *S*. *schenckii*. Both *Sporothrix* species exhibited a bilayered cell wall structure. *S*. *brasiliensis* has a thicker cell wall, which correlates with a 30% higher chitin and 100% higher rhamnose content. Variations in the cell wall architecture in culture over time are described for both species, but these changes did not correlate with significant changes in the protein or polysaccharide content. Chitin and β-glucans, but not α-glucan, were identified in the cell walls of *S*. *brasiliensis* and *S*. *schenckii*, and a conserved rhamnomannan structure was identified. This report is the first describing the cell wall architecture of *Sporothrix* species and sheds new light on the high virulence and immunopathology of *S*. *brasiliensis*.

## Introduction

Sporotrichosis, a subcutaneous mycosis that can also be present in disseminated and extracutaneous forms, was attributed for a century to a single etiological agent—*Sporothrix schenckii*. The disease was described in 1898, and the parasitic yeast phase of this thermodimorphic fungus was isolated in Brazil, in 1907 [1]. Molecular studies have since demonstrated that a complex of numerous phylogenetic species, that contains new cryptic pathogenic species [2]. This classification was later refined, and *S*. *mexicana* was placed in a separate environmental clade (the *Sporothrix mexicana* complex) [3]. *S*. *schenckii*, *S*. *brasiliensis*. *S*. *globosa* and *S*. *lurei* were recently classified in the pathogenic clade of the *Sporothrix* genus [4]. Two emerging pathogenic species, *S*. *brasiliensis* and *S*. *globosa*, therefore have significant epidemiological importance in distinct geographical regions [5–7]. Interestingly, *S*. *brasiliensis* and *S*. *schenckii*, but not *S*. *globosa*, are associated with zoonotic sporotrichosis [7, 8]. Moreover, *S*. *brasiliensis* is the causative species of the major zoonotic outbreak of sporotrichosis reported in the literature [5]. The number of cases due to cat-transmitted sporotrichosis in Brazil, only registered at the Oswaldo Cruz Foundation, is over 4000 in cats (*Felis catus*) and in humans [9], but the total number of cases is unknown and underestimated. The real numbers are even higher because sporotrichosis is not compulsorily reported in Brazil or worldwide. Additionally, cutaneous sporotrichosis can be misdiagnosed (sporotrichoid lesion/pattern) as cutaneous leishmaniasis, cutaneous tuberculosis and other cutaneous infections (http://medical-photographs.com/231-sporotrichoid-lesions.html).

In addition to its epidemiological importance, *S*. *brasiliensis* is less susceptible to the azole class of antifungals [10–12] and exhibits a higher virulence profile in a mouse model than *S*. *schenckii* clinical isolates [13, 14]. Accordingly, severe clinical cases in recent literature were attributed to *S*. *brasiliensis* infection, including fatal cases [15–17].

Very little is known about the biology of *Sporothrix* spp., and few virulence factors have been reported [18]. The genomes of *S*. *schenckii* and *S*. *brasiliensis* have 97.5% similarity [19], but evidence suggests that differences in protein expression in these fungal pathogens is significant [20]. Proteomic studies have shown that the major cell wall antigen of *S*. *schenckii*, Gp70, has a 60 kDa isoform in *S*.*brasiliensis* and is absent from non-pathogenic environmental species [20]. This evidence reinforces our hypothesis that important biological differences can exist between the pathogenic species of the genus *Sporothrix*. However, the degree of similarity of the cell surfaces of these species remains to be determined.

The cell wall is the outermost structure of fungal cells and is the first point of contact with the host upon infection and colonization. Knowledge of cell wall structure and organization that are unique to fungal pathogens is of key importance in understanding fungal pathogenesis [21]. Furthermore, understanding cell wall compositions can aid in unveiling specific mechanisms triggered by pathogen-associated molecular patterns (PAMPs) and the corresponding pathogen recognition receptors (PRRs) [22].

The cell wall of *S*. *schenckii* is composed mainly of β-glucans (1–3, 1–6, and 1–4 linkages), chitin [23] and a peptido-rhamnomannan [24], but the cell surface structure and sugar composition of other *Sporothrix* pathogenic species remain unknown. In the present work, the *S*. *brasiliensis* cell wall was studied at the biochemical and structural level and compared with that of *S*. *schenckii*. As a result, a novel cell wall model for the dimorphic fungi *Sporothrix* spp. is proposed. Cell wall structure and organization were investigated during the different growth phases of both species, as was the impact of the differences in cell wall organization on host recognition.

## Methods

### *Sporothrix* sp. strains and growth conditions

Two *S*. *schenckii* strains, ATCC MYA4820 and ATCC MYA4822, and two *S*. *brasiliensis* strains, ATCC MYA4823 and ATCC MYA4824, were used in this study. Two strains, MYA 4820 and MYA4823, are clinical isolates from the same endemic area of Rio de Janeiro State, Brazil [13].

To obtain the yeast parasitic phase, conidia of each strain were inoculated into YPD medium at pH 7.8 and grown for 7 days at 37°C under orbital agitation. A 1 mL sample of this pre-inoculum was inoculated into fresh YPD medium and cultivated for 3 to 10 days under the same growth conditions. Yeast cells were grown for either 4 or 10 days and diluted to 1 x 10^7^ cells/mL in DMEM for the macrophage interaction assays.

### High-pressure freezing transmission electron microscopy (HPF-TEM)

Samples were prepared by high-pressure freezing with an EMPACT2 high-pressure freezer and rapid transport system (Leica Microsystems Ltd., Milton Keynes, United Kingdom). After being frozen, cells were freeze-substituted in substitution reagent (1% [wt/vol] OsO_4_ in acetone) with a Leica EMAFS2. Samples were then embedded in Spurr’s resin, and additional infiltration was provided under a vacuum at 60°C before samples were embedded in Leica FSP specimen containers and polymerized at 60°C for 48 h. Semithin survey sections, 0.5 μm thick, were stained with 1% Toluidine Blue to identify areas containing cells. Ultrathin sections (60 nm) were prepared with a Diatome diamond knife on a Leica UC6 ultramicrotome and stained with uranyl acetate and lead citrate for examination with a JEOL 1400 plus transmission microscope (JEOL UK Ltd., Hertfordshire, United Kingdom) and imaging with an AMT UltraVUE camera (Deben, Suffolk, United Kingdom). The thicknesses of the inner and outer layers of the cell wall were measured using ImageJ and by averaging 100 measurements for each species (*n* = 10 cells).

### WGA, ConA and CFW fluorescence staining

Yeast cells of *S*. *schenckii* and *S*. *brasiliensis* were grown for 3–4 or 7 days in YPD broth at pH 7.8 as described above. The yeast cells were collected by centrifugation, washed with phosphate-buffered saline (PBS) and fixed with 3% formaldehyde for 15 min.The yeast cells were then washed three times with PBS and incubated in the dark for 30 min with 50 μg/mL Wheat Germ Agglutinin (WGA) FITC conjugate, 25 μg/mL Concanavalin A-(ConA) Texas Red conjugate or 25 μg/mL Calcofluor White (CFW). After being washed again with PBS, the final cell pellet was resuspended in 100 μL of PBS, and a 5 μL sample was spotted onto a poly-L-lysine microscope slide. After the sample was dried at room temperature, a drop of Vectashield (mounting medium for fluorescence) was placed on top of the sample and was covered with a coverslip to fix the sample in place. The slides were visualized in a Zeiss confocal microscope with a 63X objective. Images were processed with ZEN SP2 imaging software.

### Flow cytometry analysis

*S*. *schenckii* (MYA 4820) and *S*. *brasiliensis* (MYA 4823) were cultivated for 10 days as described above. *Candida albicans* was used as a control and was cultivated for one week in YPD. A culture sample of each strain was then submitted to a fractionation step with a discontinuous sucrose gradient (40 and 80% (w/v)) by centrifugation at 8,000 x g for 1 h at 10°C. The gradient fractions were collected by aspiration, dialyzed to remove the sucrose and fixed with 3% formaldehyde. Each fraction was labeled with 25 μg/mL of a Con A-Alexa Fluor 594 conjugate. After permeabilization of samples with 0.1% Triton X100 for 10 min and their subsequent washing, the nuclei of each fraction were labeled with 20 mM propidium iodide in DMSO. After the fractions were washed, each was resuspended in 1% formaldehyde and analyzed by flow cytometry.

Flow cytometry was performed in a MoFlo XDP apparatus (Beckman Coulter), collecting 50,000 singlet events. Fluorescence of ConA-positive events was recovered from the compensated FL3 (orange) channel using unlabeled yeast cells. PI staining was recorded in the compensated FL4 (red) channel. Total population densities were gated and analyzed using FlowJo (version 10.0.7) software.

### Cell wall fractionation

Yeast cells from cultures that were 4 days old were collected by centrifugation as described above. Briefly, the fungal pellets were suspended in distilled water with an equal volume of glass beads (0.45–0.50 mm in diameter) and shaken five times in a Braun homogenizer (Braun, Melsungen, Germany) for 1 min with cooling for 1 min on ice between shakings. Breakage of cells was monitored by light microscopy to confirm that the cells were disrupted. The homogenates were washed from the glass beads with distilled water and centrifuged at 480 x g for 5 min at 4°C. The pellet that contained the cell wall was freeze-dried and weighed. Alkali-soluble and alkali-insoluble cell wall fractions were obtained as described previously [23, 25]. Briefly, the freeze-dried material was resuspended in 1 M NaOH for 16 h, after which the suspension was centrifuged to separate the alkali-insoluble material (fraction 1) from the supernatant. The supernatant was neutralized with HCl and centrifuged, and the pellet (fraction 2) separated from the supernatant (alkali- and acid-soluble, fraction 3), which was further analyzed as previously [26–28]. To obtain rhamnomannan, we treated fraction 3 by employing Fehling´s reagent at 4°C as previously reported [23]. The insoluble copper complexes that were generated were centrifuged, washed three times with 3% KOH and twice with ethanol and collected. The resulting residue was suspended in distilled water, and cations were removed with a Dowex 50W-X4 (H^+^ form) for 1 h at room temperature; the supernatant was precipitated by the addition of 4 volumes of ethanol. The residue was collected by centrifugation at 8000 RPM for 10 min (Fraction 4, Rhamnomannan). The mother liquor of the copper complexes was neutralized with acetic acid and centrifuged. The supernatant was dialyzed for 72 h against distilled water and deionized with a mixture of Dowex 1 (HCO_3_^-^ form) and Dowex 50W-X4 (H^+^ form), the filtrate was concentrated, and polysaccharide was precipitated by the addition of 3 volumes of ethanol (Fraction 5). All obtained fractions were freeze-dried and weighed.

### Chemical analyses of cell wall fractions

To determine the sugar and total amino acid content, we analyzed all cell wall fractions as follows: for hexose content, 10 mg of each fraction sample was suspended in 1 mL of 1 M HCl, sealed in a 2 mL Wheaton 176776 ampoule and heated for 3 h at 100°C. Hydrolyzed samples were diluted 1/10 or 1/100. Quantification of sugar was conducted employing the hexose content quantification method that uses anthrone in concentrated H_2_SO_4_. To determine amino acid and amino sugar contents, we suspended 10 mg of each sample in 1 mL 6 M HCl, sealed it in a 2 mL Wheaton 176776 ampoule and heated it for 16 h at 100°C. We then used published methods as described in [26] and [27] employing alanine and glucosamine solutions as standards, respectively. For rhamnose quantification, 10 mg of fraction 3 was suspended in 1 mL of 1 M HCl, sealed in a 2 mL Wheaton 176776 ampoule, and heated for 3 h at 100°C. Hydrolyzed samples were diluted 1/10 or 1/100. Quantification of methylpentoses were conducted according to [28] using 85.7% H_2_SO_4_ and 3% cysteine in the reaction mixtures and rhamnose to construct a standard curve.

### Infrared (IR) spectroscopy

Samples were prepared as KBr pellets. IR spectra were recorded from 3500 to 500 cm^-1^ using a Nicolet iS10 IR spectrometer (Thermo Fisher Scientific, Waltham, MA, USA) coupled to OMNIC 8.0 software and following the instructions of the Infrared Spectroscopy Service, Center of Chemistry, Instituto Venezolano de Investigaciones Científicas, Caracas, Venezuela.

### Nuclear magnetic resonance (NMR) analysis

Further structural data were acquired via ^13^C- and ^1^H-NMR. The polysaccharide fraction to be analyzed (*ca*. 20 mg) and standards were dissolved in 1 mL of D_2_O and centrifuged (1000 g, 10 min), and the ^13^C spectra were obtained at 75 MHz and 70°C with a collection time of 16 h using a Bruker 300 Ultrashield spectrometer according to the instructions of the Nuclear Magnetic Resonance Service, Center of Chemistry, Instituto Venezolano de Investigaciones Científicas, Caracas, Venezuela.

### Cell wall sugar composition

Yeast cells were harvested, washed once with deionized water and disrupted in a Braun homogenizer for 5 min, alternating 1 min periods of shaking and 2 min of cooling on ice. The cell homogenate was centrifuged, and the pellet was saved and washed with 1 mM NaCl, 2% SDS and 0.3 M β-mercaptoethanol as previously described [29]. Cell wall preparations were freeze-dried before hydrolysis, and aliquots containing 5 mg were suspended in 2 M trifluoroacetic acid and incubated at 100°C for 12 h. The acid-hydrolyzed samples were analyzed by high-performance anion-exchange chromatography with pulsed amperometric detection (HPAEC-PAD) in a carbohydrate analyzer system (Dionex) equipped with an ED50 electrochemical detector with a gold electrode, a GS50 pump gradient, and a CarboPac PA10 analytical column (3 x 250 mm) with a CarboPac PA10 guard column (3 x 50 mm). Samples were eluted with a gradient of 8–20 mM NaOH with a flow rate of 0.3 mL/min for 30 min.

### Phagocytosis assay

Human monocyte-derived macrophages (hMacs) were obtained according to previous reports [30, 31]. The hMac interaction assays were performed with yeast cells of *S*. *schenckii* (ATCC MYA 4820) and *S*. *brasiliensis* (ATCC MYA 4823) grown for either 4 or 10 days, as described above. Briefly, 10^6^ peripheral blood mononuclear cells were seeded in 2 mL of supplemented DMEM containing 10% autologous human serum onto circular glass coverslips in a 24-well culture plate. The adhered monocytes were cultivated for 7 days at 37°C in an atmosphere containing 5% CO_2._

The hMacs interacted with the yeast cells of *S*. *schenckii* or *S*. *brasiliensis* at an effector: target ratio of 3:1. After 1 h of interaction, the coverslips were gently washed with PBS, pH 7.4, and stained with an Instant Prov kit (Newprov). The stained coverslips were mounted on glass slides and observed under light microscopy. The number of yeast cells inside macrophages (supplementary material) were counted. At least 50 macrophages were counted in a minimum of ten high-power fields. The number of yeasts endocytosed per macrophage was then determined.

### Ethics statement

Venous blood was collected from the cubital veins of healthy adult volunteers, and all volunteers were informed of the study before providing written consent. The number of the Certificate of Presentation for Ethical Consideration related to this study is 62785716.2.0000.5259, approved by the Ethical Committee of Hospital Universitário Pedro Ernesto, Universidade do Estado do Rio de Janeiro, Rio de Janeiro, Brazil.

### Statistical analysis

The sugar analysis and the uptake of *S*. *schenckii* and *S*. *brasiliensis* by hMac data were analyzed with a two-tailed t-test with a significance level set at p < 0.05. The program used for both statistical analyses was GraphPad Prism 5.

## Results

### Cell wall assembly of *S*. *schenckii* and *S*. *brasiliensis*

**Fig 1:**
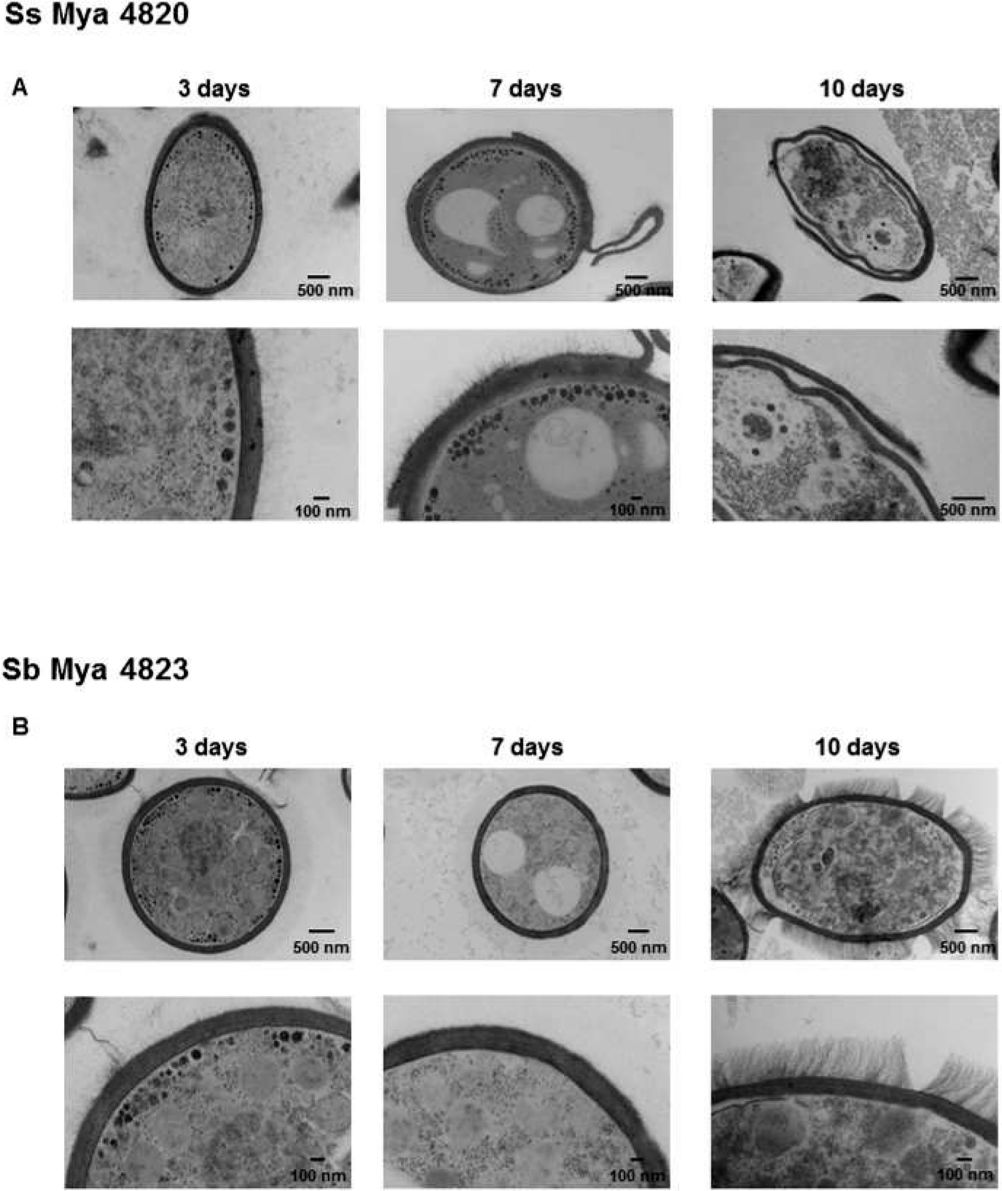
HFP-TEM of the cell wall of the yeast parasitic phase of *S*. *schenckii* (MYA4820) and *S*. *brasiliensis* (MYA4823) cultivated for 3, 7 or 10 days in YPD broth, pH 7.8. The TEM images show a cell wall double layer of *S*. *schenckii* and its shedding over time in culture (A) and a thicker and extensive microfibrillar outer layer of *S*. *brasiliensis* interconnecting yeast cells (B).

**Fig 2:**
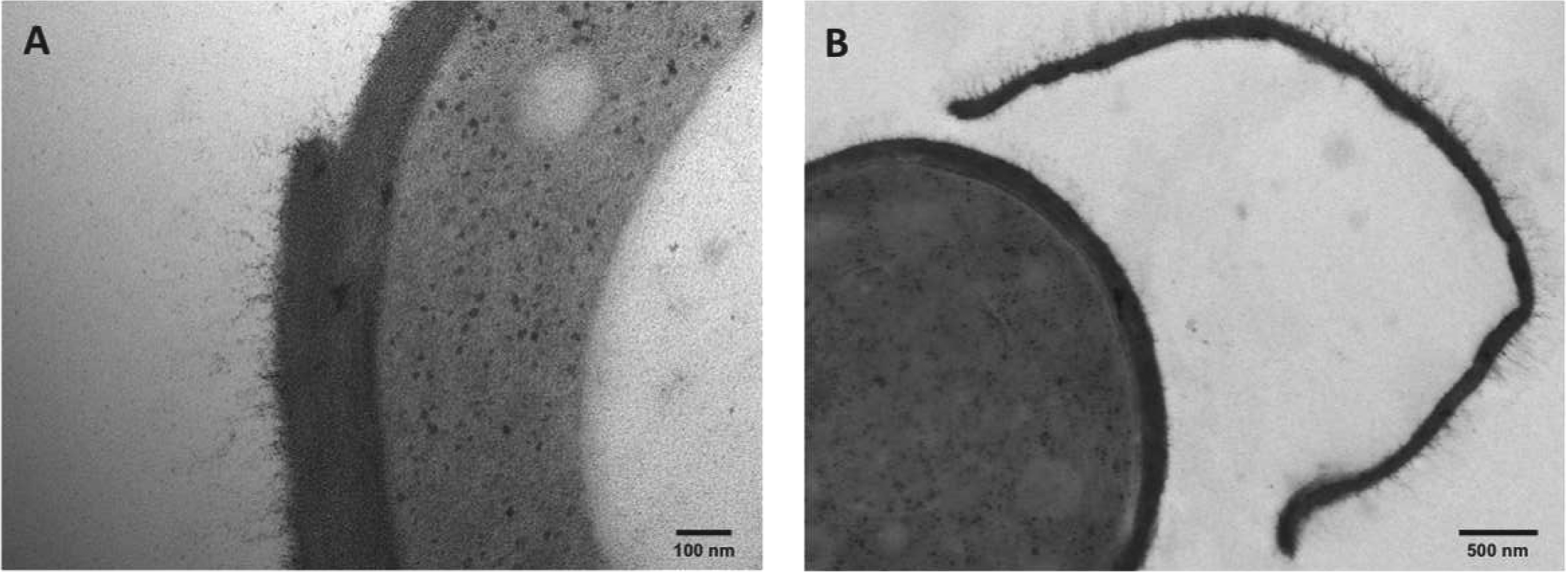
HFP-TEM of the cell wall of the yeast parasitic phase of *S*. *schenckii* MYA4822. *S*. *schenckii* yeast cells of the clinical isolate MYA 4822 were grown for 7 days in YPD broth. The TEM images show a cell wall double layer (A) and the complete shedding of a cell wall layer (B), similarly to the process observed in other *S*. *schenckii* clinical isolates (Fig 1A and S1 Fig).

The structural organization of the cell wall of *S*. *schenckii* and, for the first time, that of *S*. *brasiliensis*, was studied using HPF-TEM. The cell wall of *S*. *schenckii* was observed initially as a single layer decorated with thin fibrils in young growing cells (3–4 days in culture). However in older 7 to 10 day cultures the wall was revealed as a double-layered cell wall (Fig 1A). The cell wall thicknesses of two *S*. *schenckii* isolates were approximately 100 nm (Table 1). Interestingly, this secondary cell wall layer that appeared in older cultures of *S*. *schenckii* (stationary phase) was observed to detach entirely from the underlying cell wall via a precise fracture in the middle of the two layers (Figs 1A and 2). The same phenomenon was observed in three human clinical isolates of *S*. *schenckii* from distinct geographical regions (Figs 1A and 2 and S1 Fig).

**Table 1:** Inner layer cell wall thickness *of Sporothrix* spp. yeast cells.

	Strain ^a^	Cell wall inner layer (nm)^b^ mean ± SD
***S*. *schenckii***	**ATCC MYA 4820**	103 ± 16
	**ATCC MYA 4822**	108 + 25
***S*. *brasiliensis***	**ATCC MYA 4823**	149± 24*
	**ATCC MYA 4824**	129± 25*

a- yeast cells were grown for 3 days in YPD medium pH 7.8

b- the values corresponds to 100 measurements of distinct areas and a minimum of ten cells for each strain were analyzed.

*p < 0.05 when compared with both *S*. *schenckii* strains.

The second outer cell wall layer was shed as an intact laminate sheet (Figs 1 and 2), and intact sheets of detached cell wall were imaged in the culture fluid suspending the older cell cultures (e.g. Fig 2B). This process was observed only after 7 days in liquid culture (Fig 1A and S1 Fig) and was not correlated with any significant change in the content of structural polysaccharides (chitin and β-glucan), rhamnomannan and amino acids (Tables 2 and 3).

**Table 2:** Cell wall polysaccharide and amino acid content of *S*. *schenckii* and *S*. *brasiliensis* yeast cells at different days of growth.

Strain	Growth day	Chitin (%)	β-glucan (%)	Rhamnomannan (%)	Amino acids (%)
*S*. *schenckii* MYA 4820	4	4.73 ±0.35	20.20 ± 0.74	10.91 ± 1.10	33.99 ± 3.5
	7	6.01 ± 0.19	22.84 ±0.51	12.25 ± 0.75	36.30 ± 3.11
	10	5.91 ± 0.10	25.40 ±1.18	14.75 ± 1.07	30.82 ± 0.36
*S*. *brasiliensis* MYA 4823	4	6.11 ± 0.30*	19.12 ±1.04	12.83 ± 1.30*	35.76 ± 0.82
	7	5.86 ± 0.57	17.32 ±0.41*	13.12 ± 0.32	34.37 ± 0.93
	10	6.80 ± 0.66	17.55 ±0.55*	13.68 ± 0.50	29.55 ± 3.38

* *p* < 0.05 when compared with *S*. *schenckii* cells grown the same time

**Table 3:** Cell wall carbohydrate content in *Sporothrix* spp. yeast cell grown either 4 and 10 days in YPD broth.

	*S*. *schenckii*		*S*. *brasiliensis*	
Sugar content^a^ (%)	4 days	10 days	4 days	10 days
**Rhamnose**	17.9 ± 1.2	16.4 ± 1.1	35.4 ± 6.2*	27.7 ± 4.3*
**Glucosamine**	3.7 ± 1.0	3.4 ± 0.8	4.0 ± 0.7	3.2 ± 0.8
**Mannose**	45.1± 1.9	41.9 ± 5.9	40.2 ± 3.2	39.9 ± 0.3
**Glucose**	33.9 ± 4.1**	39.6 ± 5.7**	20.8 ± 5.5	28.2 ± 2.4

(a) Galactose was detected as trace in all the preparations.

* *p*< 0.05 when compared with *S*. *schenckii* cells grown the same time.

** *p*< 0.05 when compared with *S*. *brasiliensis* cells grown the same time.

**Fig 3:**
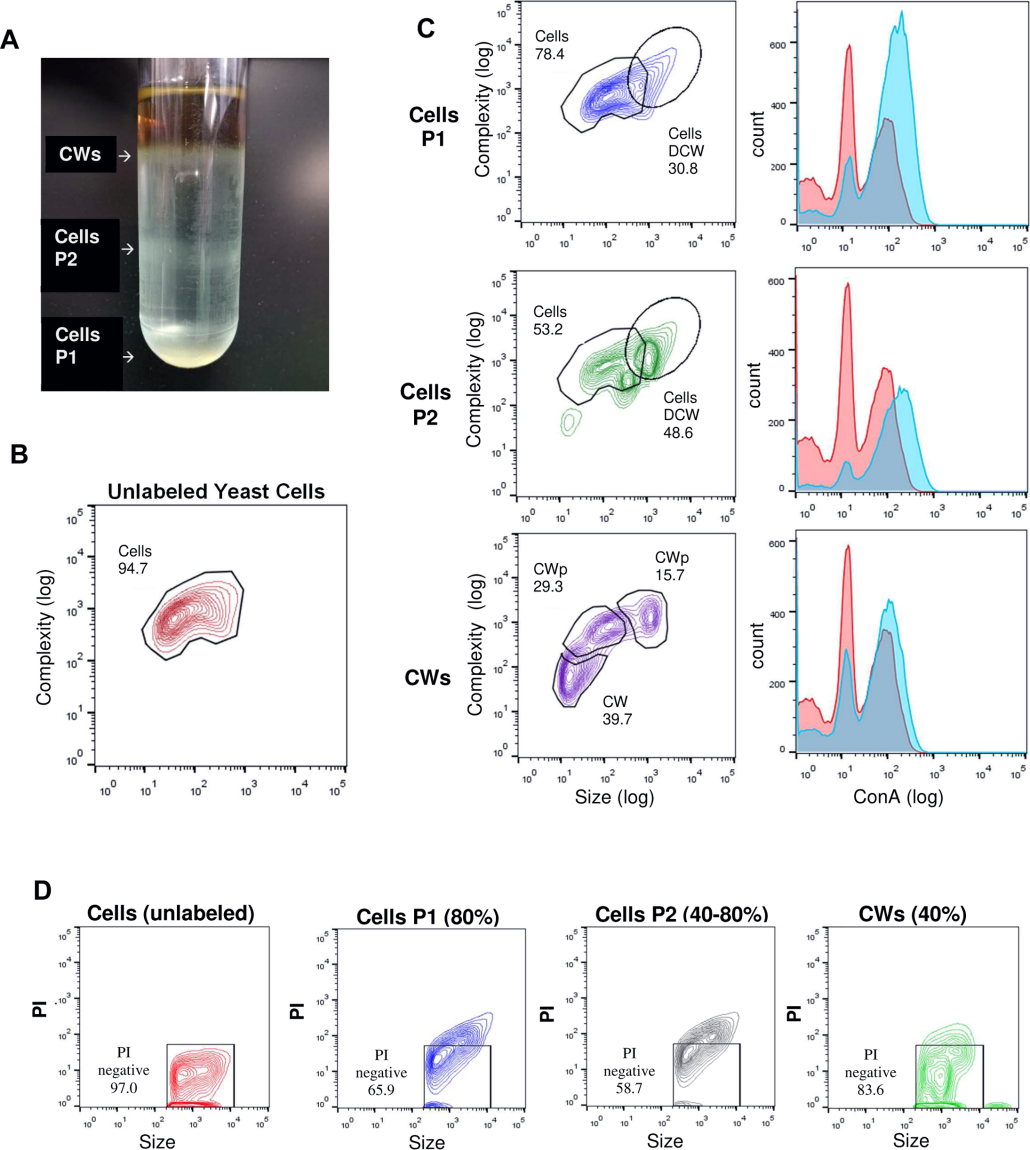
Flow cytometry analysis of *S*. *schenckii* (MYA4820 10 d) cells and cell wall fractions isolated in a sucrose gradient. (**A**) Discontinuous sucrose gradient fractionation of *S*. *schenckii* (MYA4820) after 10 days in culture (yeast phase). (**B**) Unstained whole cells (10 days culture) were analyzed to compensate for autofluorescence and to locate the cell populations. (**C**) Gradient fractions were analyzed by size and complexity (forward scatter vs. side scatter) and by ConA-Alexa Fluor 594 labeling. The red histogram represents the fluorescence pattern of unstained whole cells, and the blue histogram represents the fluorescence pattern for the labeled ConA-Alexa Fluor 594 gradient fraction; (**D**) flow cytometry analysis of Triton X-100-permeabilized sucrose gradient fractions stained with propidium iodide (PI) to assess the number of cells (via DNA labeling) present in the sample. Size vs. PI staining is plotted. PI fluorescence was used to detect nuclei (Cells P1 and P2) and nucleic acid contaminated cellular debris (CW particles).

To confirm that complete cell wall detachment occurred, *S*. *schenckii* yeast cells were isolated from cultures that were 10 days old and fractionated in a discontinuous sucrose gradient (40–80% sucrose) (Fig 3A) Three fractions were collected and labeled with ConA-Alexa Fluor and propidium iodide (PI) to detect PRM and nucleic acids, respectively. For DNA detection, all fractions were permeabilized before PI staining, as detailed in Methods. Fractions from the gradient flow cytometry analysis (Fig 3) revealed the presence of two cell populations (cells P1 and cells P2) by their size and granularity (complexity) features and the presence of isolated cell walls (gradient top) These observations were corroborated by ConA-Alexa Fluor 594 staining (Fig 3C) and PI nuclei staining of the different fractions (Fig 3D). Cell walls exhibited a distinct granularity and traces of PI staining compared to both P1 and P2 fractions suggesting that the observed pattern was due to cell wall compaction and contamination with nucleic acid debris after its detachment from the cell membrane. Two clear *S*. *schenckii* cell populations were identified and denoted as cells with a double cell wall (Cells-DCW); single cell wall (CW) fractions.

**Fig 4:**
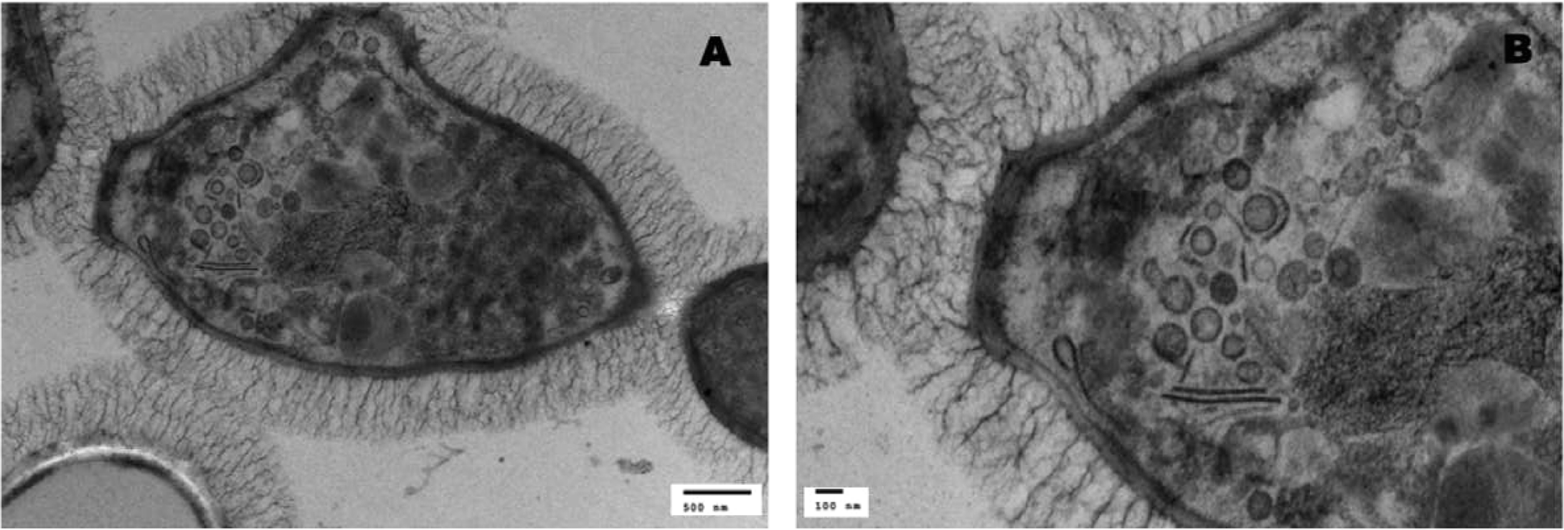
HFP-TEM of the cell wall of *S*. *brasiliensis* yeast parasitic phase. (A, B) HFP-TEM images at two magnifications show details of long cell wall fibrils (~400 nm) in *S*. *brasiliensis* (10 days in culture) interconnecting the yeast cells. In (B) the fibrils form a physical bridge between two *S*. *brasiliensis* yeast cells. Scale bars are indicated in each panel.

*S*. *brasiliensis* had a thicker inner cell wall layer than *S. schenckii* (Table 1) when grown under the same culture conditions, and cells had an impressive 400 nm outer fibrillar layer that could be clearly observed in yeasts at later stages of growth (Fig 1B and Fig 4). Differences in the inner cell wall layer appeared to be species-specific as indicated by the analysis of two strains of each species (Table 1). On average, *S*. *brasiliensis* cell walls were 20 to 40% thicker than *S schenckii* cell walls but this difference was dependent on the isolate (Table 1).

Chitin, β-glucan, rhamnomannan and amino acid content and cell wall sugar composition did not change markedly in either *S*. *brasiliensis* or *S*. *schenckii* between 4 and 10 days in culture (Tables 2 and 3). However, the cell wall of *S*. *brasiliensis* has 30% more chitin content and 100% more rhamnose content than that of *S*. *schenckii* (Tables 2 and 3). HPF-TEM showed that the cell wall fibrils of *S*. *brasiliensis* sometimes formed bridges between yeast cells (Fig 1B and Fig 4).

**Fig 5:**
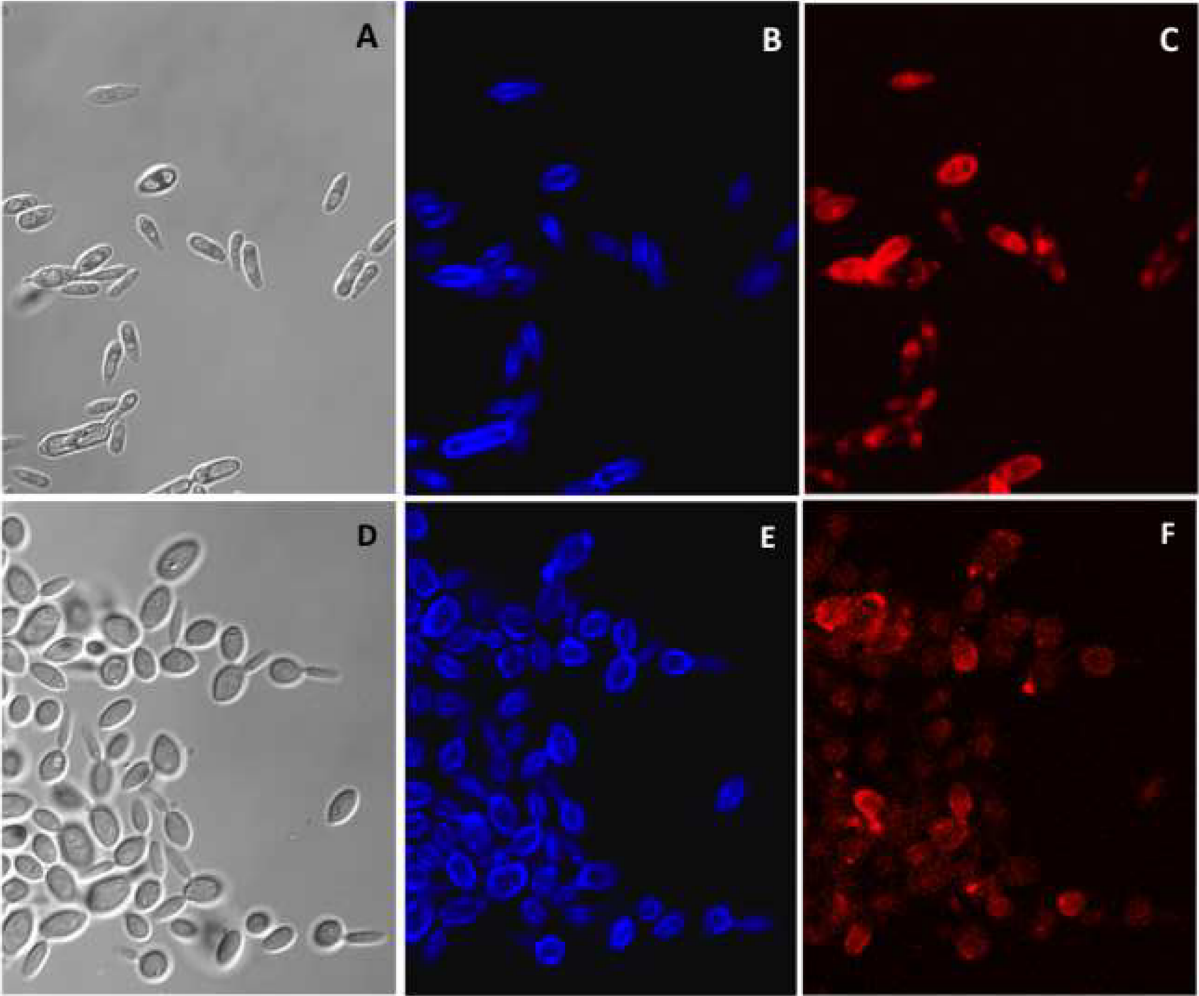
Fluorescence microscopy of *Sporothrix* spp. 4 days yeast cells labeled with ConA and CFW. *S*. *schenckii* (A to C) and S. *brasiliensis* (D to F) yeast cells were grown for 4 days in YPD broth (exponential growth phase). The yeast cells were labeled with Calcofluor White (B and E) and Con A-Texas Red (C and F). Differential interference contrast (DIC) images of *S*. *schenckii* and *S*. *brasiliensis* are shown in panels A and D, respectively. The labeled yeast cells were visualized in a Zeiss Confocal Microscope (63X magnification).

**Fig 6:**
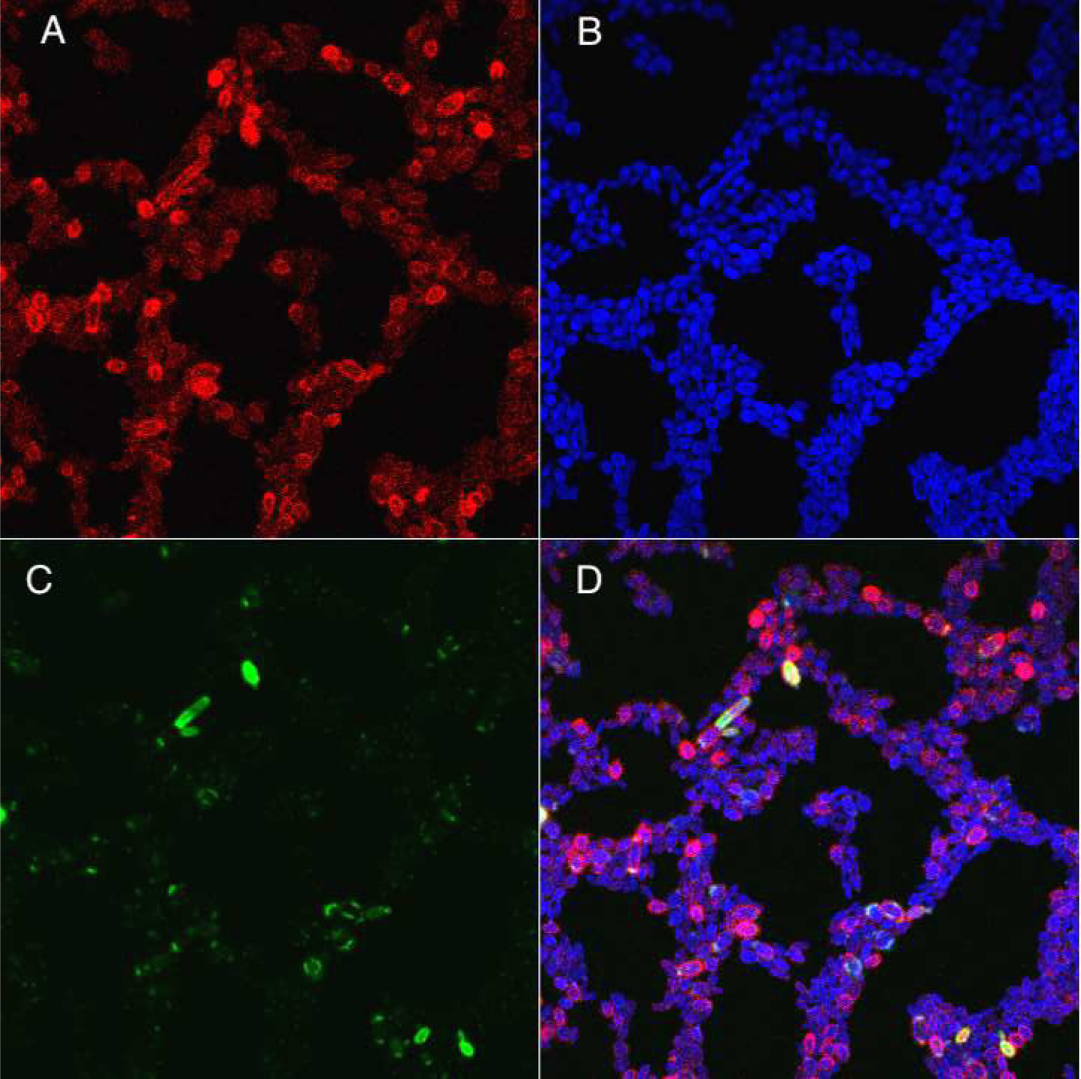
Fluorescence microscopy of *S*. *brasiliensis* 7 days yeast cells labeled with ConA, WGA and CFW. Yeast cells were grown for 7 days in YPD broth (stationary growth phase) and labeled with Con A-Texas Red (A), Calcofluor White (B), and WGA-FITC (C), and the images were overlaid (D). The labeled yeast cells were visualized in a Zeiss Confocal Microscope (63X magnification).

Confocal microscopy of 4 days yeast cells labeled with ConA and CFW showed a positive PRM and chitin for both *Sporothrix* species (Fig 5). The ConA fluorescence pattern observed for *S*. *schenckii*, in contrast with that of *S*. *brasiliensis* (Figs 5A and 6F) had had a more uniform labeling pattern on the yeast cell surface (Fig 5C). Fc-dectin-labeling of exposed β-1,3 glucan was negative for both species. *Sporothrix* spp. is a highly pleomorphic fungus and some differences in the yeast cell shape and size were expected for each batch of yeast cells [13].

Confocal images of 7 days yeast cell suggested that these cell wall bridges of *S*. *brasiliensis* could potentially form a network of yeast cells that adhered to each another (Fig 6). Similar networks of cells were not formed by *S*. *schenckii* under the same experimental conditions. Additionally, *S*. *brasiliensis* yeast cells were ConA and CFW positive. A non-uniform punctate distribution of ConA red fluorescence was observed on the surface of the yeast cells of *S*. *brasiliensis*. Few yeast cells were positive for WGA, indicating no chitin exposure in the native cell wall (Fig 6C and 6D).

### Polysaccharide structure of *S*. *brasiliensis*

For the structural analysis of polysaccharides, the cell walls of *S*. *schenckii* and *S*. *brasiliensis* cells in the yeast phase were purified and fractioned by the alkali solubility method for fungal cell wall analyses, as described in the Methods section. Structures were characterized by infrared (IR) spectroscopy as well as by ^1^H-NMR and ^13^C-NMR. The ^1^H-NMR and ^13^C-NMR spectra were compared with spectra that were previously reported for *S*. *schenckii* [32–36].

**Fig 7:**
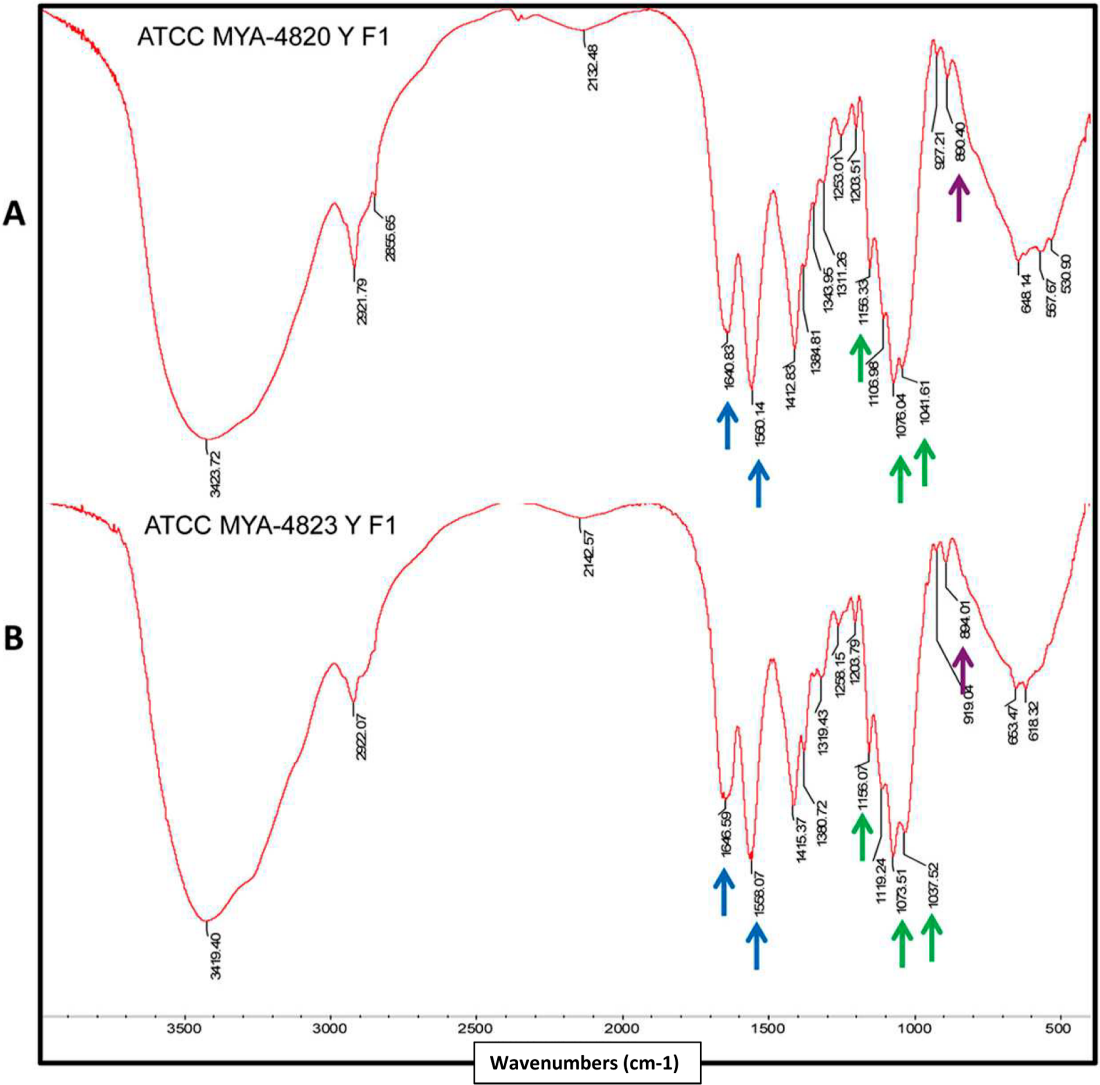
Infrared spectroscopy of the alkali-insoluble cell wall fraction of *S*. *schenckii* (MYA 4820) and *S*. *brasiliensis* (MYA 4823). Infrared spectra of *S*. *schenckii* (A) and *S*. *brasiliensis* (B) cell wall showing the peaks at energies corresponding to the cell wall polysaccharides chitin (1560 and 1640 cm^-1^; blue arrows) and β-glucan (890 cm^-1^; purple arrow). The presence of β-glucans (1→3 and 1→6) are evidenced by peaks at 1156, 1076, and 1041 cm^-1^ (green arrows).

IR spectra of the alkali insoluble cell wall fraction exhibited absorption signals characteristic of chitin and β-glucans (Fig 7), including a strong, wide band at approximately 3400 cm^-1^ and additional bands at approximately 2920 and 1412 cm^-1^ [37]. Absorption bands at approximately 1560 and 1640 cm^-1^ are evidence of the presence of chitin, while the presence of glucan with a β-configuration was evident because of an absorption band at approximately 890 cm^-1^ (Fig 7A and 7B). Additionally, the presence of absorption peaks belonging to β-(1,3)-(1,6)-glucan (at 1156, 1076 and 1041 cm^-1^) are present in the IR spectra of both species [37]. α-Glucans, distinctly from other dimorphic fungi, were not found in the *Sporothrix* spp. cell wall [36].

**Fig 8:**
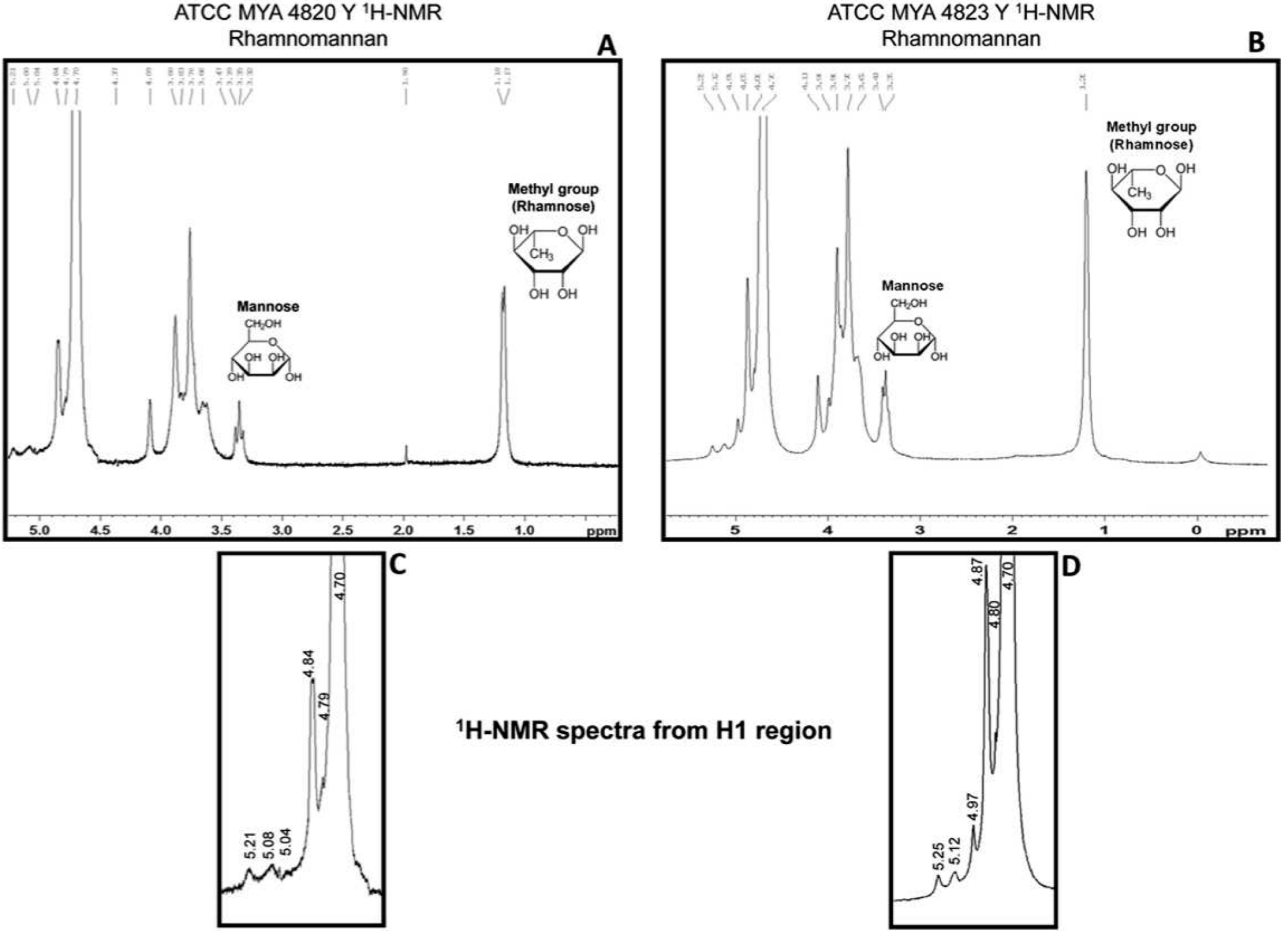
^1^H-NMR of the rhamnomannan fraction isolated from *S*. *schenckii* and *S*. *brasiliensis* yeast cells. ^1^H-MNR spectra showing the signals corresponding to the α-L-Rha 1→3 α-D-Man*p* structure (A and B). The H1 region of the rhamnomannans of *S*. *schenckii* (C) and *S*. *brasiliensis* (D) is enlarged.

The rhamnomannan fraction isolated from the alkali-soluble fraction, as described In Methods, was further analyzed by nuclear magnetic resonance. The general pattern of ^13^C-NMR spectra for both species displayed signals corresponding to a rhamnomannan structurally similar to that described for the yeast phase of *S*. *schenckii*, and whose main chain or backbone is composed of mannose units linked by α-1,6 glycosidic bonds with single units of rhamnose as side chains (Table 4 and S2 Fig). ^1^H-NMR spectra for both species are shown in Fig 8A and 8B. We focused on the H1 region (Fig 8C and 8D), which has been previously used to identify rhamnomannan from *S*. *schenckii* [33]. Analysis of the complete ^1^H-NMR spectra for both species (Fig 8A and 8B) indicated the presence of a methyl group because of three protons linked to the C of the methyl group that are represented at chemical shifts between 1.17 and 1.20 ppm; this methyl group belongs to rhamnose, as it is the only monosaccharide present in rhamnomannan that possesses this chemical structure. Additionally, signals are also found that correspond to a proton linked to carbon 5 (3.5–3.39 ppm) near the methylene group from carbon 6 in the mannose ring (Fig 8A and 8B).

**Table 4:** *S*. *schenckii* and *S*. *brasiliensis* rhamnomannan ^13^C-NMR signals, yeast phase.

Species (strain)	Structure	^13^CNMR-signal, δ_c_ (70°C) (ppm)*						
		C1	C2	C3	C4	C5	C6	CH_3_
*S*. *schenckii* (MYA 4820)	α-L-Rhamnopyranose nonreducing end units	96.2	70.4	N.R^#^.	71.9	68.7	——	16.7
	3,6-di-O-substituted α-D-mannopyranose units	99.4	65.9	74.9	64.6	70.1	N.R.	——
*S*. *brasiliensis* (MYA 4823)	α-L-Rhamnopyranose nonreducing end units	96.3	70.4	N.R.	71.9	68.7	——	16.7
	3,6-di-O-substituted α-D-mannopyranose units	99.4	65.9	74.9	N.R.	70.8	N.R.	——

*Signals assigned following Gorin *et al*., 1977

# N.R.—No ressonance signal detected.

The presence of proton signals at 5.21–5.25, 5.08–5.12 and 4.84–4.87 ppm are characteristic of *S*. *schenckii* rhamnomannan, as previously reported [33, 35]. However, an extra signal (4.97 ppm) was only present in *S*. *brasiliensis* rhamnomannan (Fig 8D) and was not related to any previous reported result.

### Glycogen alpha-particles are found near the plasma membrane and cell wall of *Sporothrix* sp. yeast cells

The supramolecular organization of glycogen was recently determined in mammals [38]. Liver glycogen is composed of beta-particles (~20 nm), which form larger clusters linked by hydrogen bonds called glycogen alpha-particles. These alpha-particles have a characteristic rosette structure evidenced by TEM [37–39]. The glycogen alpha-particles measure at least 50 nm and can reach 300 nm [39, 40].

**Fig 9:**
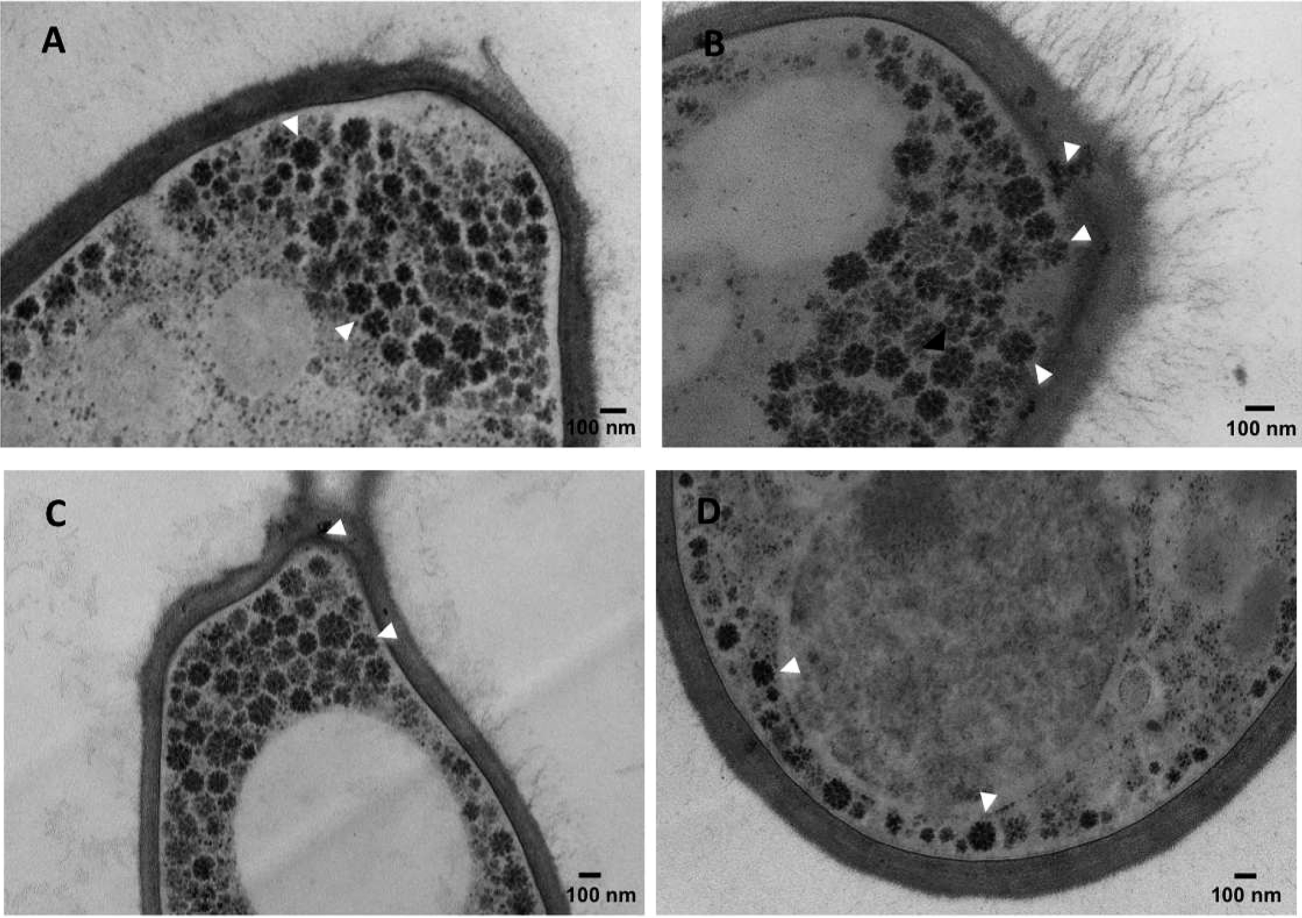
HFP-TEM images showing the typical rosette-like glycogen alpha-particles in *Sporothrix* sp. yeast cells. TEM images show rosette-like glycogen alpha-particles organized close to the plasma membrane and cell wall of (A and B) *S*. *schenckii* (MYA4820) and (C and D) *S*. *brasiliensis* (MYA4823). The arrowheads indicate rosette-like glycogen alpha-particles.

We report the presence of the characteristic rosette-like structures in dimorphic fungus *Sporothrix* spp., indicating glycogen alpha-particles (Fig 8 and S3 Fig). The glycogen alpha-particles in *S*. *schenckii* and *S*. *brasiliensis* yeast cells were consistently distributed around the plasma membrane and close to the cell wall (Fig 1 and Fig 9, S3 Fig). Most glycogen alpha-particles were localized at the budding poles of yeast cells (Fig 9C). The glycogen alpha-particles disappeared in both species after 7 and/or 10 days in culture (Fig 1A and 1B).

### Interaction of *S*. *schenckii* and *S brasiliensis* with human macrophages

**Fig 10:**
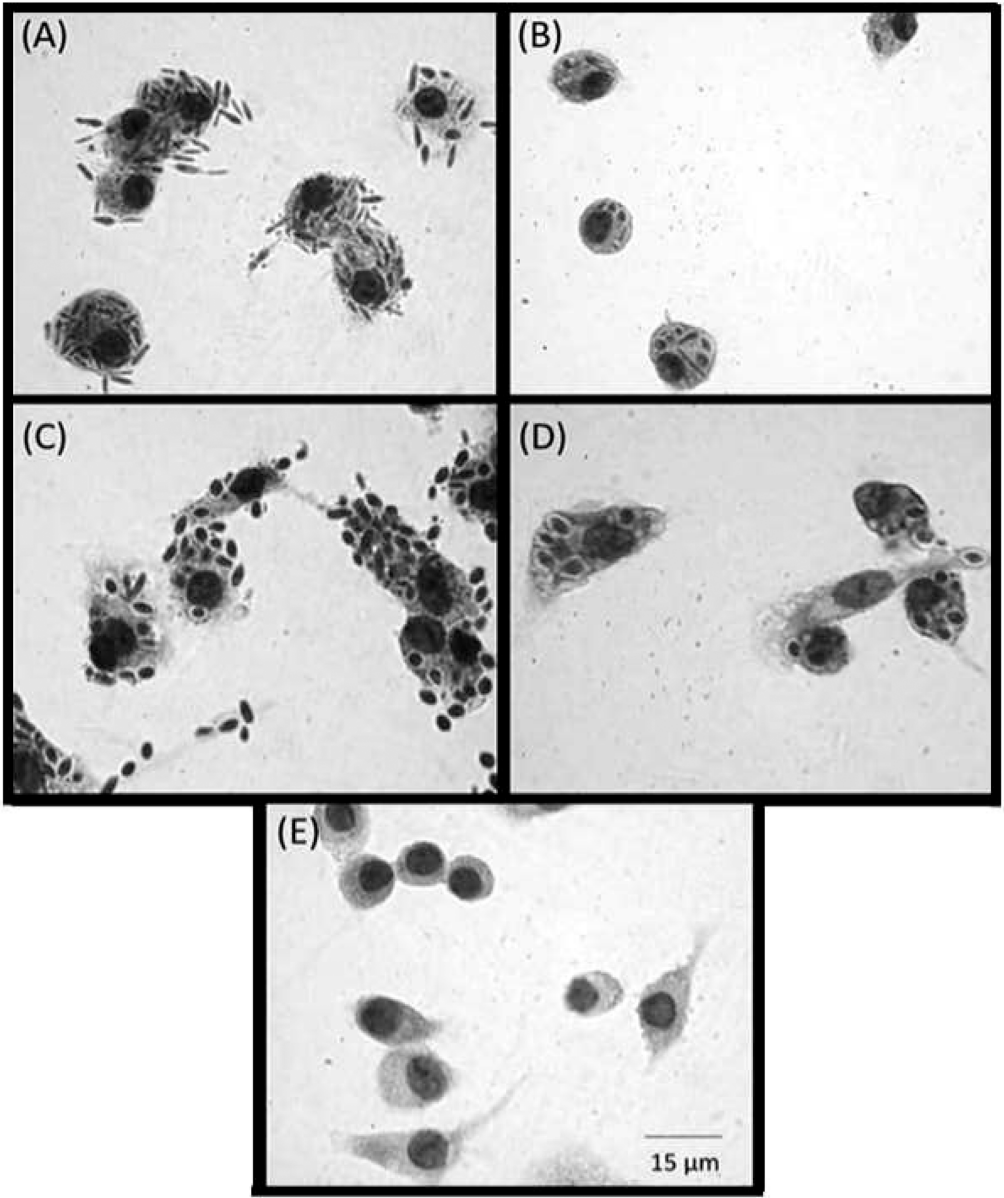
Panel illustrating the uptake of *Sporothrix* spp. by hMac. Optical microscopy images showing the differences in uptake by human monocyte-derived macrophages of *Sporothrix* sp. yeast cells cultivated for 4 or 10 days in YPD broth. *S*. *schenckii* is shown at 4 days (A) and 10 days (B). *S*. *brasiliensis* is shown at 4 days (C) and 10 days (D). (E) hMac uninfected control. The slides were stained with an Instant Prov Kit (Newprov) to determine the internalization of yeast cells.

**Fig 11:**
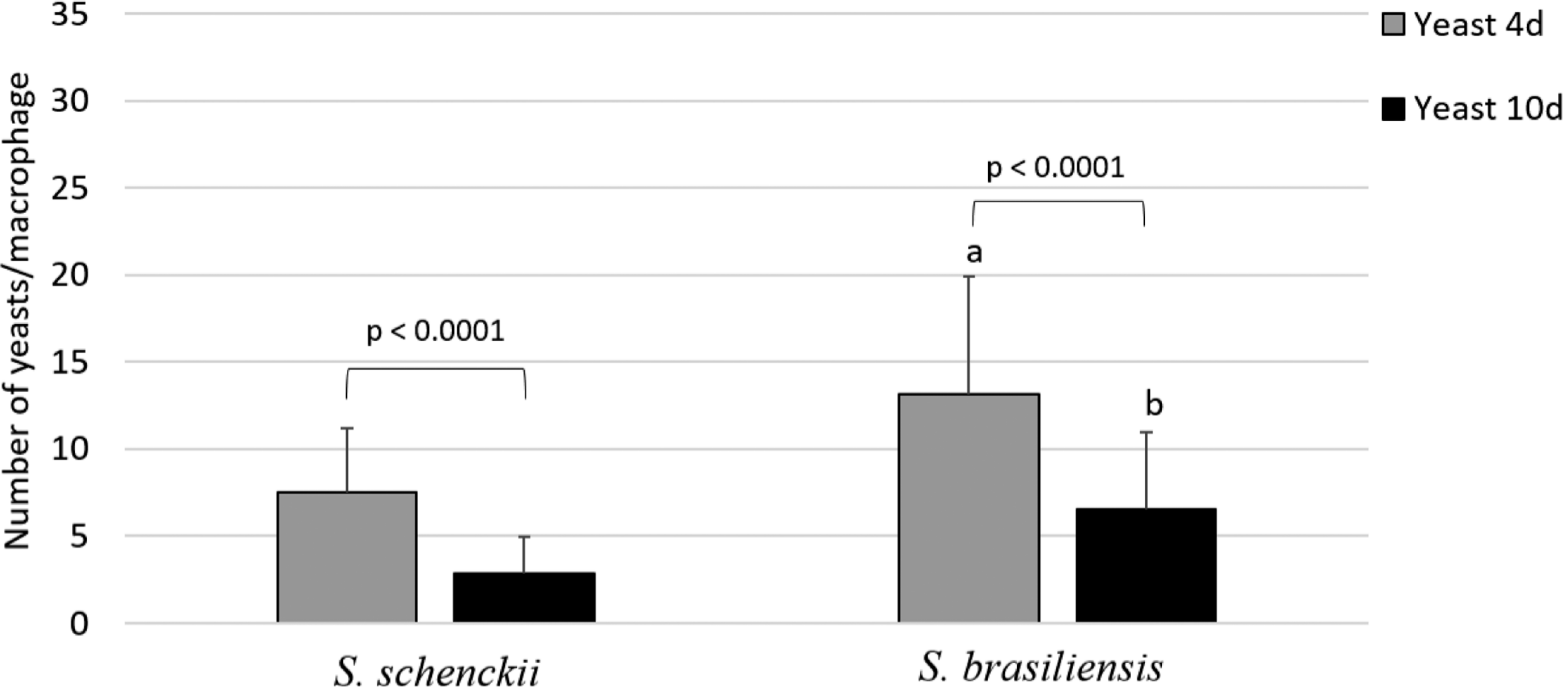
Uptake by human monocyte-derived macrophages (hMac) of *S*. *schenckii* (MYA 4820) and *S*. *brasiliensis* (MYA4823) yeast cells cultivated for 4 and 10 days in YPD broth. The uptake of *S*. *schenckii* and *S*. *brasiliensis* yeast cells (4 and 10 days) are reported as the mean values of three experiments performed in duplicate with their standard deviation (SD). Unpaired t-test: (a) *p* ≥ 0.0001 *S*. *brasiliensis* compared with *S*. *schenckii*– 4 days yeast cells; (B) *p* ≥ 0.0001 *S*. *brasiliensis* compared with *S*. *schenckii*– 10 days yeast cells.

Differences in the wall architecture and composition of pathogenic fungi impact host recognition by innate immune cells [22]. To correlate the differences observed on the cell walls of *S*. *schenckii* and *S*. *brasiliensis* with host recognition (innate immune system), we determined the uptake of yeast cells by human monocyte-derived macrophages. Fig 10 and Fig 11 shows that phagocytosis of *S*. *brasiliensis* yeast cells was significantly greater than that of *S*. *schenckii* yeast cells. The impact of cell wall modifications was observed in culture over time (Fig 1), and hMac phagocytosis of yeast cells of both species was much less when cells were grown for 10 days (Fig 10 and Fig 11) than when grown for 4 days. These results suggest that differences in the composition (Tables 2 and 3) and/or architecture of the cell surface (Fig 1) during yeast growth might lead to differences in recognition of the fungus by professional phagocytes. The cell wall is a dynamic structure that can vary according to environmental conditions, which leads us to conclude that the cell wall plasticity observed for *Sporothrix* spp. can also occur inside the host.

## Discussion

Cell wall glycoconjugates of pathogenic fungi are involved in virulence and pathogenicity and can modulate the innate immune response [21, 22, 40]. The present study shows that *Sporothrix* spp., especially *S*. *schenckii*, can slough off cell wall layers, which have the potential to cause antigenemia or inflammation at a distance from the site at which the mother pathogen cell is located. The most common lymphocutaneous form of sporotrichosis is characterized by the migration of the pathogen by the lymphatic vessels, but sporotrichosis can also spread in the manner observed in disseminated cutaneous and extracutaneous forms [1].

Some structural cell wall glycoconjugates, chitin and β-1-3 and β-1-6-glucans, are found in pathogenic and non-pathogenic species and are involved in the innate immune response [41–42]. These glycoconjugates are known as PAMPs. The exposure of chitin and β-glucans on the fungus surface favors their binding to the corresponding PRRs exposed by the host cells, allowing the uptake of the microorganism and/or triggering the secretion of specific cytokines [41, 42]. In addition to these common structural polysaccharides, other homo- and heteropolymers and/or mannoproteins are present on the surface of fungal pathogens and can be very specific for certain genera or species [22, 43–45]. A typical example is the expression of α-glucans that was described to occur on the surface of dimorphic fungi. These polymers play an important biological role by protecting the fungal cell of the dimorphic fungi *Paracoccidioides brasiliensis*, *Histoplasma capsulatum* and *Blastomyces dermatitidis* from the host immune response by preventing the exposure of β-glucans that are recognized by the dectin-1 receptor [22]. Furthermore, α-glucans were also found in the pathogenic species of the *Scedosporium/Pseudallescheria* complex [45]. We show here that α-glucans are not present on the cell surfaces of the dimorphic fungi *S*. *schenckii* and *S. brasiliensis*. Furthermore, *S. brasiliensis*, which is a highly virulent emerging species in the *Sporothrix* pathogenic clade [13, 14] and is related to cat-transmitted sporotrichosis [5, 9], had a higher chitin and rhamnose contents and, thicker cell wall than *S*. *schenckii*.

Cell wall peptido-rhamnomannans were first described on the surface of *S*. *schenckii* and *Ceratocystis* species [24, 43, 44]. The PRMs of *S*. *schenckii* consist of a peptide core adorned with *O*- and *N*-linked chains [34, 43, 44]. PRMs were later found in other pathogenic species of the *Scedosporium/Pseudallescheria* complex that bear epitopes similar to those of *S*. *schenckii* [44–49]. The main epitope described in the *N*-linked chains of *S*. *schenckii* yeast cell PRMs was the α-L-Rha*p* 1→3 α-D-Man*p* side chain [43, 47] and is also present in the PRM O-linked chains [34]. The most important epitopes described in all human pathogenic species presenting PRMs on their surface were subsequently related to the *O*-linked chains, which exhibit important biological functions [44, 45, 49]. The *O*-linked chains of *S*. *schenckii* PRMs were characterized as bearing the cell wall ConA-binding sites of α-Man*p* 1→2 α-Man*p* linked to Ser/Thr and presenting the antigenic epitopes of α-L-Rha*p* 1→3 α-D-Man*p*, α-L-Rha*p* 1→4 α-D-GlcA*p* and α-L-Rha1→4 [α-L-Rha*p* 1→2] α-D-GlcA*p* [34, 44]. These three epitopes were present in the *O*-glycosidically linked tri-, tetra- and pentasaccharides, respectively [49]. The ConA reactivity reported for the PRM is absent in the rhamnonannan fraction isolated by alkali extraction (N-linked chains) [50], In the present work, we used confocal fluorescence microscopy to demonstrate surface mannan evidenced by positive ConA cell surface reactivity of not only *S*. *schenckii* yeast cells but also *S*. *brasiliensis*. We also identified a rhamnomannan moiety on the *S*. *brasiliensis* yeast cell surface that has a repeating α-D-Man*p* 1→6 α-D-Man*p* 1→ backbone with α-L-Rha*p* 1→3 α-D-Man*p* side chains. Our ^13^C-NMR results suggest that the structure of this cell wall polysaccharide is conserved in the yeast phase of both *S*. *schenckii* and *S*. *brasiliensis*.

Although we could not find major differences in the structure of rhamnomannans (free polysaccharides or PRM *N*-linked chains) at the biochemical level, an extra resonance signal was present in the H1 region (at 4.97 ppm) of the ^1^H-NMR spectra in *S*. *brasiliensis* samples but was absent in *S*. *schenckii* spectra. This signal was previously observed in the 1970s [33, 35] in the so-called type II rhamnomannan, and this polymer was only found in a few isolates of *S*. *schenckii* [43]. The rhamnose content of *S*. *brasiliensis* is 100% higher than that of *S*. *schenckii*. The former species also has 30% more chitin and significantly more rhamnomannan than that latter (Tables 2 and 3). The higher content of these cell wall components correlates with the differences observed between these two species at the ultrastructural level.

HPF-TEM data show that two *S*. *brasiliensis* strains (MYA 4823, a feline clinical isolate, and MYA 4824, a human clinical isolate) have a thicker cell wall than two *S*. *schenckii* strains (MYA 4820 and MYA 4822, human clinical isolates). *S*. *schenckii* and *S*. *brasiliensis* isolates exhibited low and high virulence profiles, respectively [13, 14]. A fibrillar material was present on the outer layers of both species, a length of up to 400 nm. The fibrils were more clearly observed in the older cells of both species and, although not statistically significant, the rhamnomannan content was slightly higher in yeast cells kept in culture for 7 and 10 days (Table 2). The ConA reactivity observed by confocal microscopy corroborates this model, indicating that PRM is present in the outermost cell wall layer since the ConA epitopes have been described to occur in only PRMs [50]. Our data suggest that the longer fibrils were associated with significantly higher rhamnose and rhamnomannan content of *S*. *brasiliensis* yeast cells.

The cell wall architecture in both species differed greatly, and the cell wall differences became clearer as yeast cells aged. The conditions associated with longer term culture (starvation, cell density dependent changes in physiology, etc.) induced the formation of a second cell wall layer in *S*. *schenckii*. This layer had been observed by electron microscopy previously but was attributed to a cell wall fracture [51] and, later, to an unknown cell wall-shedding mechanism in *S*. *schenckii* [52]. We show here for the first time that *S*. *schenckii* produces a second cell wall layer in culture, using cryo-fixation methods that rapidly immobilizes the cells and preserves their fine structure of cells with much greater fidelity than conventional fixation protocols. Furthermore, we clearly show that by an unknown process, this second layer can become detached from the outer cell surface and delivered into the extracellular milieu (S1 Fig). In contrast, older *S*. *brasiliensis* yeast cells were shown by confocal microscopy to have an extensive outer cell wall fibrillar layer that was more electron-dense than those of *S*. *schenckii*. and that formed interconnecting bridges between yeast cells (Fig 4) to form organized cell clusters (Fig 6). These differences in cell wall architecture in the parasitic phase of *S*. *schenckii* and *S*. *brasiliensis* can suggest explanations for differences observed in the uptake of each species by human macrophages. The uptake rate was significantly different between yeast cells cultivated for 4 and 10 days and correlated with the cell wall modifications resulting from longer times spent in culture (Fig 1). Our group demonstrated in a murine model of sporotrichosis that *S*. *brasiliensis* is more virulent than *S*. *schenckii* [13]. We also demonstrated that *S*. *brasiliensis* strains frequently exhibit drug resistance to itraconazol [11] and to miltefosine [12]. The drug resistance of microorganisms is currently related to, among other factors, their capacity to form biofilms [53, 54]. Interestingly, we observed that *S*. *brasiliensis* fibrils interconnected yeast cells forming cell clusters. The capacity of this species to form a mature biofilm that could alter its drug resistance profile needs further investigation.

Glycogen, an intracellular storage polysaccharide, is a complex branched polymer of glucose units that can assemble into a number of morphologies that include supramolecular clusters of beta-particles that form larger clusters of alpha-particles. These alpha-particles have a characteristic rosette-like structure [37–39], and are related to a distinct physiological function in mammalian cells [39, 55] [38]. Glycogen rosettes that have a similar appearance to mammalian glycogen alpha-particles have also been observed in plants [56, 57]. In the present work, we used HFP-TEM to show the well-organized presence of glycogen alpha-particles around the plasma membrane of *Sporothrix* spp. and close to the cell wall. Our hypothesis, that is being investigated in an ongoing project, is that these particles serve as a source of glucose for cell wall enzymes. We found a great number of glycogen alpha-particles at the budding pole of yeast cells, where intense *de novo* synthesis of cell wall components occurs (Fig 9). A previous report showed that a polysaccharide-rich particulate fraction could be isolated from cytoplasmic extracts of *C. albicans* and that those polysaccharide particles were similar to those of rabbit liver glycogen [58]. The polysaccharide particles identified in *C*. *albicans* have the macromolecular structure that is characteristic of glycogen, and they appear as rosette-like structures in electron microscopy images [39, 58]. The presence of rosette-like structures around the plasma membrane of *C*. *albicans* was also observed in HFP-TEM preserved preparations [Neil Gow, personal communication].

**Fig 12:**
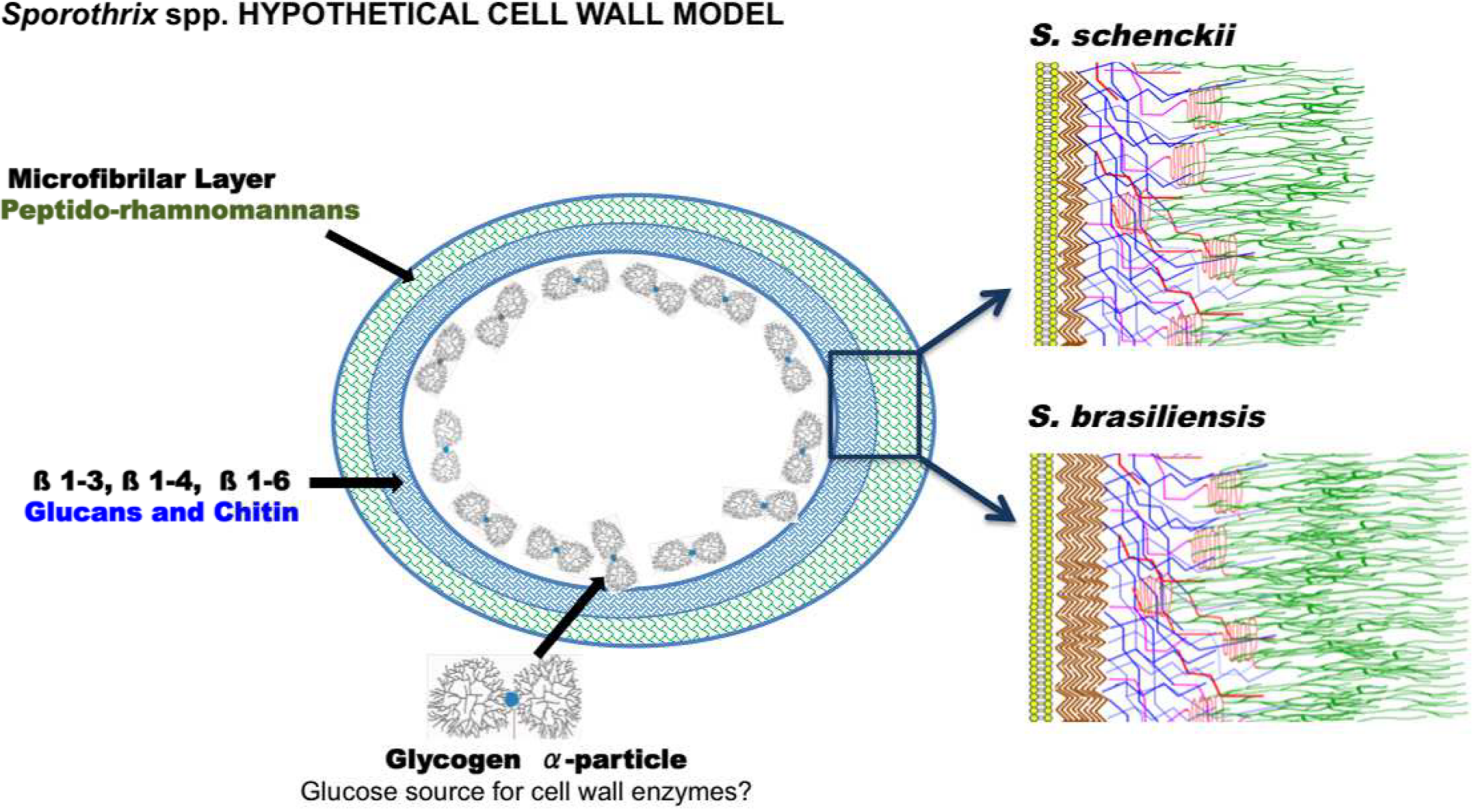
A new hypothetical cell wall model for the dimorphic pathogenic fungi *S*. *schenckii* and *S*. *brasiliensis*. This model is based on biochemical and structural analyses (IR, ^1^H-NMR and ^13^C-NMR spectroscopy) as well as HPF-TEM and fluorescence microscopy with ConA-Texas Red, WGA-FITC and CFW, which indicated the presence of a peptido-rhamnomannan component localized on the outermost fibrillary layer (green); chitin (brown) and β-glucans 1→3 (blue) and 1→6 (pink) β-glucans at the innermost layer; and rosette-like glycogen alpha-particles organized close to the plasma membrane and cell wall. α-Glucans, whose presence has been described in other dimorphic pathogenic fungi, were absent. The presence of rhamnomannan polymers and the absence of α-glucans (based on IR spectra) on the cell surface support the proposition of a new model for the thermodimorphic fungi of the genus *Sporothrix* (Fig 10). Other models proposed for pathogenic dimorphic fungi [reviewed by 23] differ significantly with the particular biochemical and structural characteristics observed for the cell wall of *S*. *schenckii* and *S*. *brasiliensis*.

We propose a new cell wall model for *S*. *schenckii* and *S*. *brasiliensis* based on the data presented here (Fig 12). Our model includes the presence of PRMs on the outermost cell wall layer as observed in the confocal images with ConA. The ConA reaction is related to PRM sites, where ConA binds [34, 44, 50]. Additionally, the higher chitin content of *S*. *brasiliensis* is illustrated in addition to its longer outer fibrillar layer. Chitin is proposed to be localized in the inner layer of the cell wall, as WGA-negative labeling indicates that this structural polysaccharide is not normally exposed on the yeast cell surface of *Sporothrix*. Rosette-like structures characteristic of glycogen alpha-particles organized around the plasma membrane is also illustrated in both species.

## Supporting information

S1 Fig HFP-TEM showing a double cell wall layer in several clinical isolates of *S*. *schenckii*. The yeast parasitic phase of the clinical isolates MYA 4820 (A), MYA 4821 (B and C) and MYA 4822 (D) were cultivated for 7 days in YPD broth. The TEM images show the formation of a double cell wall layer in all *S*. *schenckii* clinical isolates. Scale bars are indicated in each panel.
(TIF)

S2 Fig ^**13**^**C-NMR spectroscopy of rhamnomannan from *S*. *schenckii* (panel A) and *S*. *brasiliensis* (panel B).** Spectra were obtained at 75 MHz with a collection time of 16 h and at 70°C using a Bruker 300 Ultrashield spectrometer. Signals corresponding to α-L-rhamnopyranose nonreducing end units and 3,6-di-O-substituted α-D-mannopyranose units were assigned according to Gorin et al., 1977 (Table 4).
(TIF)

S3 Fig Presence of rosette-like glycogen alpha-particles surrounding the plasma membrane of *Sporothrix* spp. HFP-TEM images showing the presence of glycogen alpha-particles (typical rosette-like structures) well-organized close to the plasma membrane of two clinical isolates of *S*. *schenckii* (MYA4822) and *S*. *brasiliensis* (MYA4824).
(TIF)
