# Supplementary figures and images for: Cell walls of the dimorphic fungal pathogens Sporothrix schenckii and Sporothrix brasiliensis exhibit bilaminate structures and sloughing of extensive and intact layers
Source: PLoS Negl Trop Dis. 2018 Mar 9;12(3):e0006169. doi: 10.1371/journal.pntd.0006169 (PMC5903669; doi:10.1371/journal.pntd.0006169)

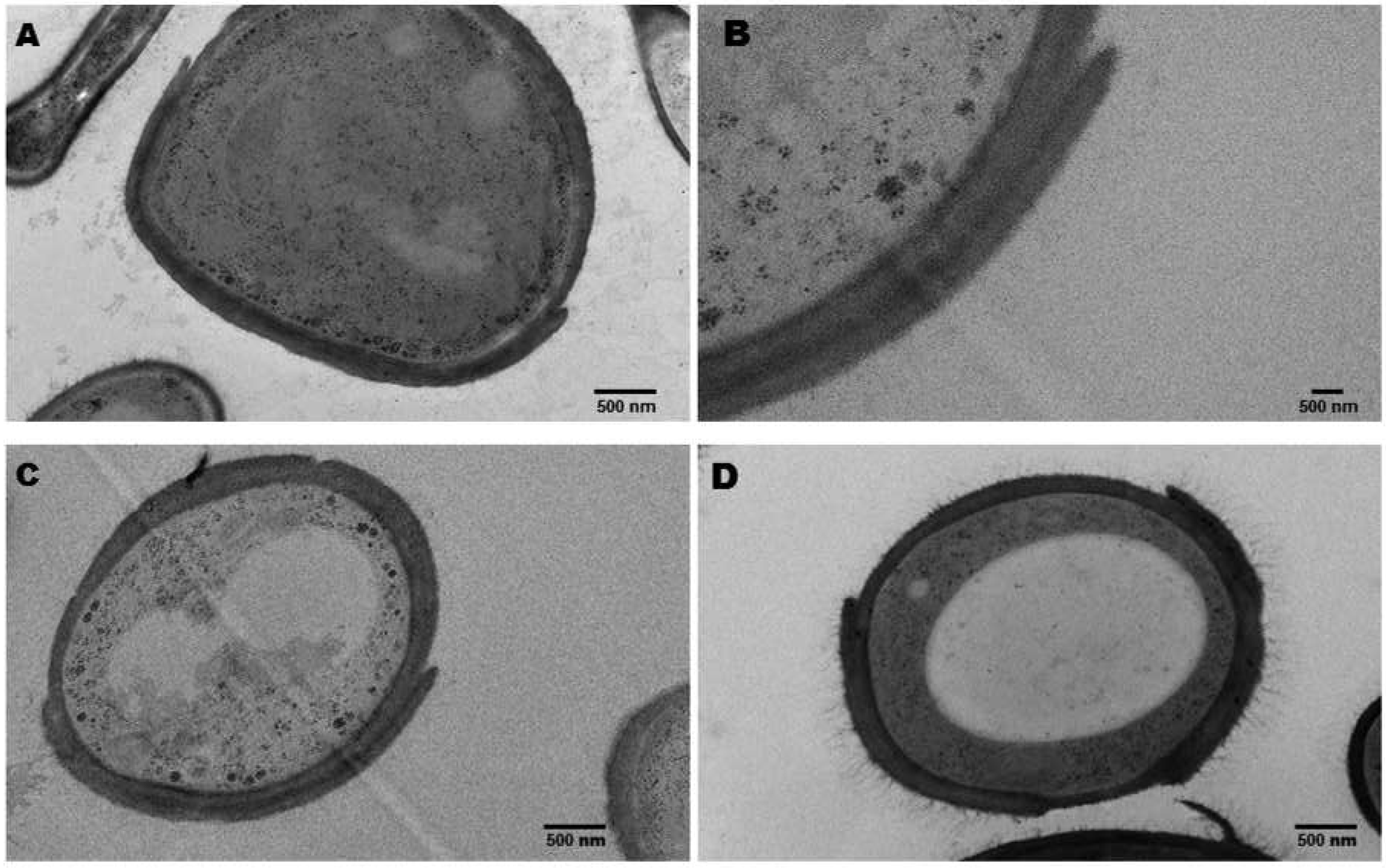

Supplement: S1 Fig — The yeast parasitic phase of the clinical isolates MYA 4820 (A), MYA 4821 (B and C) and MYA 4822 (D) were cultivated for 7 days in YPD broth. The TEM images show the formation of a double cell wall layer in all S. schenckii clinical isolates. Scale bars are indicated in each panel. (TIF) [file pntd.0006169.s001.tif]

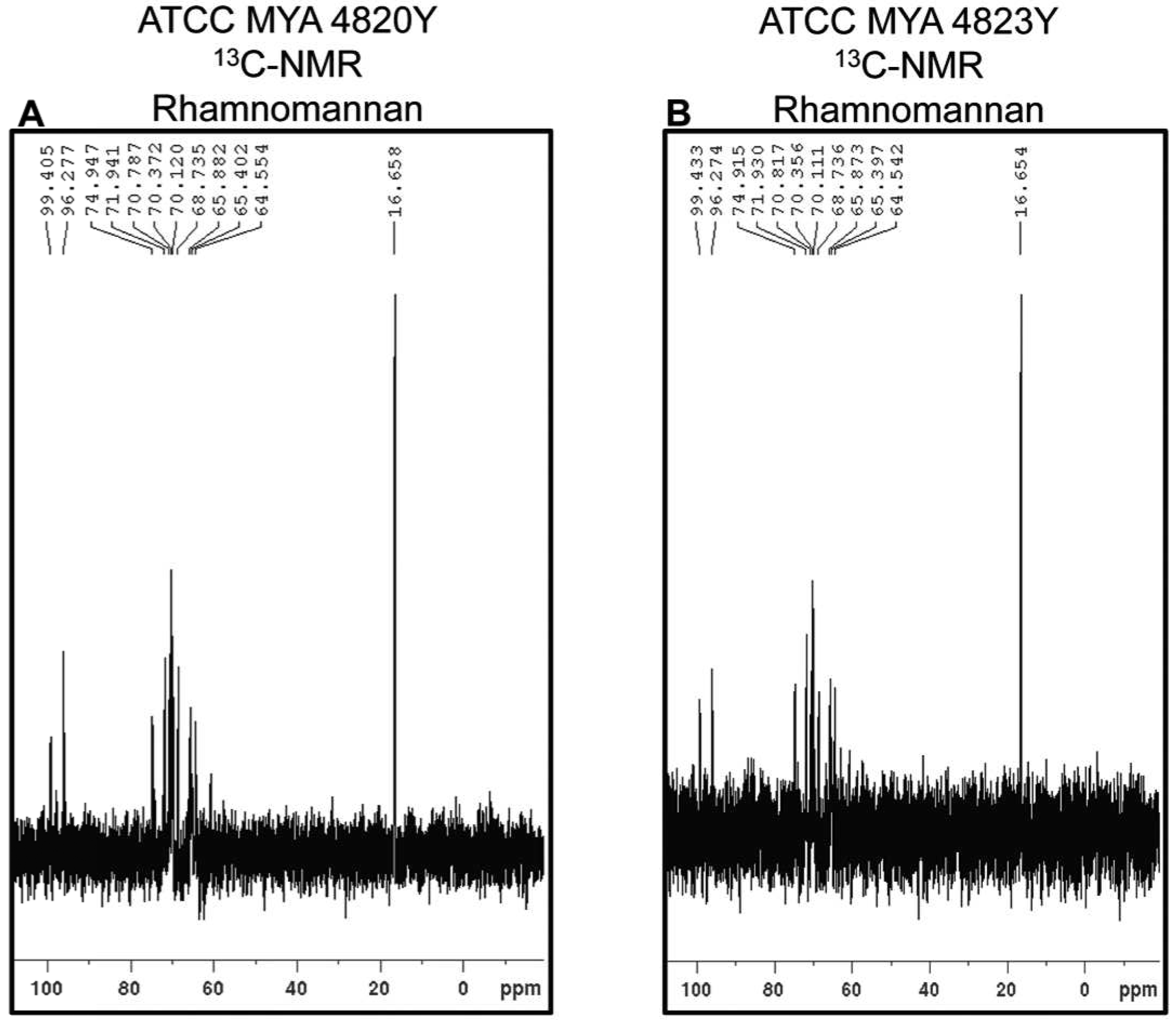

Supplement: S2 Fig — 13C-NMR spectroscopy of rhamnomannan from S. schenckii (panel A) and S. brasiliensis (panel B). Spectra were obtained at 75 MHz with a collection time of 16 h and at 70°C using a Bruker 300 Ultrashield spectrometer. Signals corresponding to α-L-rhamnopyranose nonreducing end units and 3,6-di-O-substituted α-D-mannopyranose units were assigned according to Gorin et al., 1977 (Table 4). (TIF) [file pntd.0006169.s002.tif]

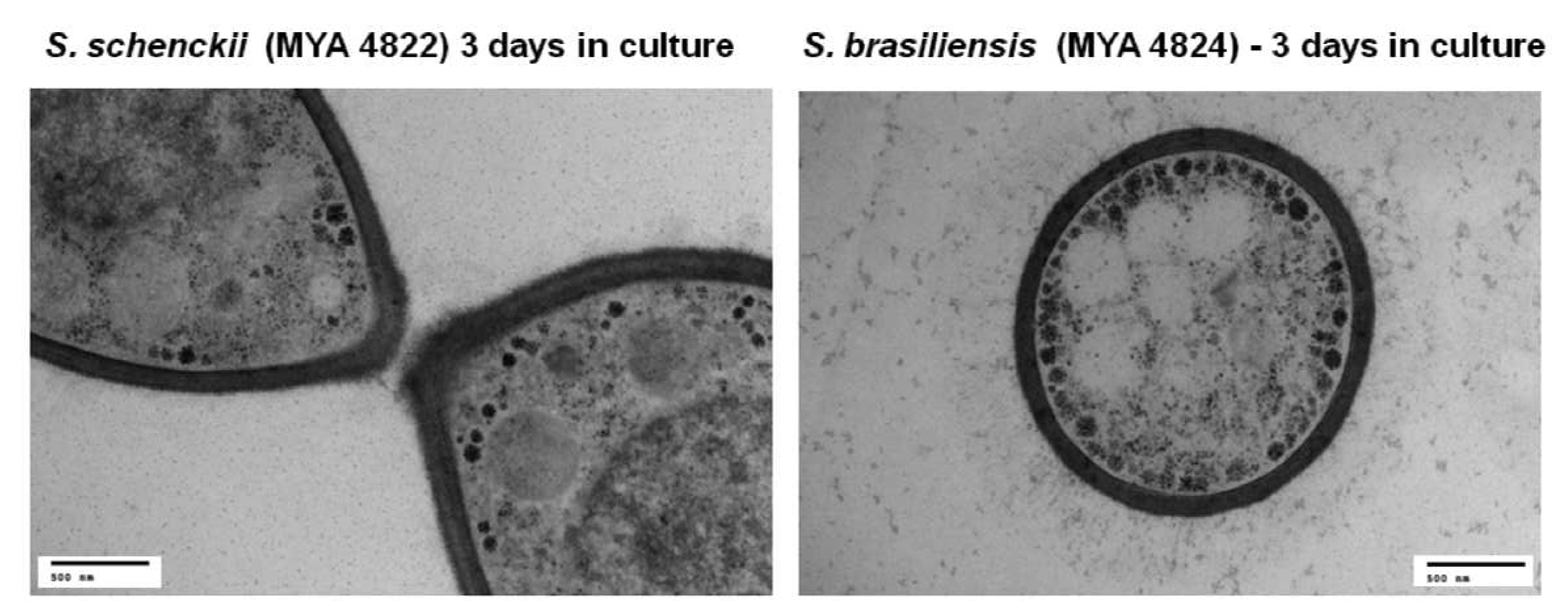

Supplement: S3 Fig — HFP-TEM images showing the presence of glycogen alpha-particles (typical rosette-like structures) well-organized close to the plasma membrane of two clinical isolates of S. schenckii (MYA4822) and S. brasiliensis (MYA4824). (TIF) [file pntd.0006169.s003.tif]
